# Exploring the Potential of Microservices in Internet of Things: A Systematic Review of Security and Prospects

**DOI:** 10.3390/s24206771

**Published:** 2024-10-21

**Authors:** Abir El Akhdar, Chafik Baidada, Ali Kartit, Mohamed Hanine, Carlos Osorio García, Roberto Garcia Lara, Imran Ashraf

**Affiliations:** 1 LTI Laboratory, ENSA, Chouaib Doukkali University, El Jadida 24000, Morocco; elakhdar.abir@ucd.ac.ma (A.E.A.); baidada.c@ucd.ac.ma (C.B.); alikartit@gmail.com (A.K.); hanine.m@ucd.ac.ma (M.H.); 2 Universidad Europea del Atlántico, Isabel Torres 21, 39011 Santander, Spain; carlos.osorio@unini.edu.mx (C.O.G.); roberto.garcia@unini.edu.mx (R.G.L.); 3 Universidad Internacional Iberoamericana, Campeche 24560, Mexico; 4 Universidade Internacional do Cuanza, Cuito EN 250, Angola; 5 Universidad de La Romana, La Romana 22000, Dominican Republic; 6 Department of Information and Communication Engineering, Yeungnam University, Gyeongsan 38541, Republic of Korea

**Keywords:** Internet of Things, microservices, potential security, standard security, systematic review

## Abstract

With the rapid growth of Internet of Things (IoT) systems, ensuring robust security measures has become paramount. Microservices Architecture (MSA) has emerged as a promising approach for enhancing IoT systems security, yet its adoption in this context lacks comprehensive analysis. This systematic review addresses this research gap by examining the incorporation of MSA in IoT systems from 2010 to 2024. From an initial pool of 4388 studies, selected articles underwent thorough quality assessment with weighted critical appraisal questions and a defined inclusion threshold. This study represents the first comprehensive systematic review to investigate the potential of microservices in IoT, with a particular focus on security aspects. The review explores the merits of MSA, highlighting twelve benefits, eight key challenges, and eight security risks. Additionally, the eight best practices for implementing MSA in IoT systems are extracted. The findings underscore MSA’s utility in fortifying IoT security while also acknowledging complexities and potential vulnerabilities. Moreover, the study calls attention to the importance of incorporating complementary technologies including blockchain and machine learning to address identified gaps effectively. Finally, we propose a taxonomic classification for Microservice-based IoT security patterns, facilitating the categorization and organization of security measures in this context. Such a review can help researchers and practitioners identify existing gaps, highlight potential research directions, and provide guidelines for designing secure and efficient microservice-based IoT systems.

## 1. Introduction

“Things can tell you much more than people and can talk constantly” stated IoT expert Dr. Timothy Chou in his latest book [1], highlighting the significant potential of the Internet of Things in benefiting humanity. To structure this potential, he proposed a five-layer hierarchy for classifying devices based on their possible uses: do, learn, collect, connect, and things. The term Internet of Things refers to a broad range of items equipped with sensors and actuators that gather, process, and exchange data with other items, software, and platforms. The IoT architecture, named also the IoT technology stack, has a variety of designs. The three most popular and widely used designs in business, industrial research, and applications are the three-layer [2,3]; the four-layer [4], which is the most common one and involves the perception layer, the network layer; the middleware layer, and the application layer, and the five-layer [5] structures. Another recent study identifies up to 12 layers [6]. With greater granularity in IoT systems, featuring additional layers and independent components, the attack surface expands, increasing vulnerability to security threats. This complexity introduces more entry points and interfaces, each representing a potential security risk. Securing each layer and its interactions is critical. The diverse technologies across layers often have unique security requirements, calling for tailored approaches to each. As IoT systems grow more complex, security strategies must evolve to manage the increasing number of components and their interactions, minimizing vulnerabilities. As the IoT revolutionizes technology interaction, enabling seamless communication and connectivity between devices and the digital world, the rising number of connected devices and data volumes has thrust IoT into the forefront of businesses and industries worldwide. Despite its immense promise, significant challenges persist concerning security [7] and performance. In response, microservices architecture emerges as a promising solution for IoT development.

Microservices architecture structures a software application as a collection of loosely coupled, independently deployable services. Each service is designed to perform a specific business function and communicates with other services through well-defined APIs. Microservices’ unique characteristics offer a modular, flexible, and decentralized approach [8] that empowers organizations to construct scalable, resilient, and highly available applications. Within the IoT context, the security of data and system integrity is a paramount concern due to the vast number of interconnected devices and the sensitive nature of the data they handle. Microservices offer a multitude of advantages, including bolstered security and amplified performance [9]. Their distributed nature can potentially enhance security by allowing for more granular security policies at the component level and reducing the risk of single points of failure, thereby enhancing the fortification of IoT systems. Moreover, microservices deliver a scalable and distributed data processing paradigm that drives superior performance, minimizing latency if designed well, and optimizing response times for IoT systems. It is important to note that, in some cases, Microservices Architecture (MSA) can introduce latency due to the increased communication overhead between services. However, with careful implementation strategies such as efficient caching, asynchronous communication, and appropriate network configurations, these challenges can be effectively managed, leading to an overall robust system. Practical applications of MSA in IoT include smart cities, where microservices enable the efficient management of urban resources like traffic control and energy distribution, and connected healthcare systems, where patient data from various medical devices are securely processed and analyzed in real time. In industrial IoT, MSA enhances the management and monitoring of connected machinery and devices across large-scale industrial networks, improving operational efficiency and system security. Nevertheless, the integration of microservices in IoT also presents challenges [10], including complex system monitoring and management due to their distributed nature, necessitating specialized skills and tools for effective development and deployment. Consequently, prudent evaluation of microservices’ benefits and drawbacks becomes imperative before adopting this approach within the IoT landscape.

Despite the substantial body of work exploring microservices and IoT independently, there is a notable lack of studies that integrate these domains with a focus on security. While some prior reviews have addressed security-related literature for microservice-based systems [11,12], as well as IoT systems [13,14], we identified a lack of systematic reviews specifically focusing on the security aspects of MSA-based IoT systems. Notably, a related work closer to our objectives is a survey discussing microservices in IoT security [15]. Another significant study provides a comprehensive review of the state-of-the-art in microservice-based IoT systems [16], focusing on how microservices architecture can enhance non-functional characteristics such as reliability and availability. This review identifies the strengths, weaknesses, and opportunities of microservices in IoT systems, and emphasizes the need for further research to address existing challenges in ensuring reliability and availability. To address this research gap, the primary objective of this work is to systematically explore the potential benefits of adopting microservices in IoT and assess how this approach can enhance both the security and performance of IoT systems. This study not only consolidates existing research but also extends knowledge by the following:

1. Identifying Key Security Challenges: analyzing how microservices address security challenges in IoT systems.
2. Assessing Current Solutions: evaluating the effectiveness of existing microservice-based solutions in enhancing IoT security.
3. Highlighting Research Trends: using bibliometric analysis to uncover trends, popular research topics, and influential papers in this domain.
4. Proposing Future Directions: suggesting potential areas for future research based on identified gaps and trends.

Through a comprehensive analysis of existing literature, this work will provide insights into the benefits, limitations, and best practices associated with microservices adoption in the context of IoT. By examining solution proposals, case studies, empirical research, and industry trends, this study should provide a detailed understanding of the potential advantages and challenges of microservices in IoT, as well as the implications for practitioners and researchers. Ultimately, this review aims to fill the void in systematic research and contribute to the development of best practices and guidelines for the adoption of microservices in IoT, enabling organizations to build secure, scalable, and high-performance IoT systems.

In Section 2, an overview of the search techniques and approaches utilized in this paper is provided, along with a detailed description of the review process and the research questions posed. The ensuing Section 3 outlines the principal findings uncovered through this comprehensive study, including the challenges and security risks faced by MSA IoT systems and the architectural solutions provided by microservices. Moreover, this section delves into emerging research trends, best practices, and future directions for the integration of microservices in IoT software, accompanied by pertinent suggestions for researchers. Section 4 offers a forward-looking perspective on the subject matter while highlighting potential research gaps by proposing a taxonomic classification of security patterns for MSA IoT systems. In Section 5, potential threats to the validity of this research are discussed. Finally, concluding remarks are presented in Section 6.

## 2. Research Protocol

In this study, we utilized the PICOS [17] framework, which stands for population, intervention, comparison, outcome, and study design, to define the research questions. By applying the PICOS framework, the research questions were ensured to be well-structured, focused, and aligned with the specific goals of the study, facilitating a comprehensive and systematic approach to evidence-based inquiry.

- Population (refers to the group or system being studied): IoT systems;
- Intervention (refers to the specific procedure or action being investigated): microservices for IoT;
- Comparisons (involve the alternative conditions or groups against which the intervention is evaluated): standard IoT architectures (including other software architectures)
- Outcomes (refer to the specific effects or results that the study aims to measure): the security benefits of adopting microservices in IoT systems
- Study design (refers to the type and methodology of studies included in the review): focus on studies that provide solutions, methodologies, security mechanisms, or other procedures to handle microservice-based IoT systems.

### 2.1. Research Questions

RQ1: What is the annual count of publications in the research domain of microservices for IoT?

This question seeks to identify the number of research publications that have been produced each year in the field of microservices for IoT. Knowing the annual publication rate in this research area helps researchers and practitioners assess the interest and growth of research in this domain.

RQ2: What are the primary publication outlets for research in the field of microservices for IoT?

This question investigates the primary sources for research publications in the area of microservices for IoT. Knowing the leading research venues in this field can help researchers identify the key contributors, topics of interest, and emerging trends in the field of microservices for IoT.

RQ3: What are the benefits of adopting microservices in IoT systems, and how do these benefits compare to other software architectures?

This research question seeks to explore the advantages of using microservices in IoT systems and compare them to other software architectures. It aims to identify what benefits can be gained by adopting microservices, such as improved performance, scalability, and security.

RQ4: What are the key challenges in adopting microservices in IoT systems, and how can they be overcome?

This research question seeks to identify and address the obstacles and difficulties that hinder the adoption of microservices in IoT systems. Possible challenges could include issues related to performance, security, interoperability, complexity, or resource constraints. The research may involve developing strategies, techniques, or best practices to mitigate or resolve these challenges, evaluating the effectiveness of existing solutions, or proposing new approaches to overcome the identified obstacles.

RQ5: What are the security risks associated with using microservices in IoT systems, and how can these risks be mitigated?

This research question explores the potential security risks associated with using microservices in IoT systems. It aims to identify common vulnerabilities, threats, and attack vectors as well as examine strategies that can be used to mitigate them.

RQ6: What are the performance implications of adopting microservices in IoT systems, and how can these be measured and optimized?

This research question focuses on the performance implications of using microservices in IoT systems. It aims to identify how microservices affect system performance and examine approaches for measuring and optimizing system performance.

RQ7: How can microservices architecture be implemented in IoT systems, and what are the best practices to follow?

This research question focuses on the practical aspects of implementing microservices in IoT systems. It aims to identify the most effective ways to design and deploy microservices in IoT systems and identify best practices that can help ensure success.

RQ8: What are the most promising future directions for research in the area of microservices adoption in IoT systems?

This research question aims to explore the potential future trends and directions for the adoption of microservices in the context of IoT systems. It could involve identifying new technologies or techniques that could enhance the efficiency or scalability of microservices in IoT, investigating the integration of microservices with emerging IoT platforms or architectures, or exploring the potential benefits of incorporating microservices in specific IoT use cases.

### 2.2. Search Techniques

Systematic reviews rigorously synthesize existing research on a specific topic using various search techniques to identify relevant studies. The most common method (query-based method) involves searching electronic databases with predefined search terms, which is effective but may miss studies with different terminologies or in unsearched databases. Venue-based techniques focus on specific journals or conference proceedings, identifying key studies within a field but potentially missing important research outside these sources. Snowballing, which involves reviewing references of identified studies [18], is useful for finding related research but can be time-consuming and may not capture all relevant studies. Other techniques, such as hand-searching reference lists, contacting experts, and reviewing grey literature, have their own limitations, including time constraints, potential bias, and quality control issues.

To address these limitations, we propose a hybrid methodology using multiple search techniques to carefully assess the quality and relevance of each study included in this review and eventually ensure a comprehensive search. The proposed hybrid methodology includes the following:

- Venue-based search;
- Query-based search;
- Snowballing.

### 2.3. Search Process

#### 2.3.1. Methodology

**Figure 1:**
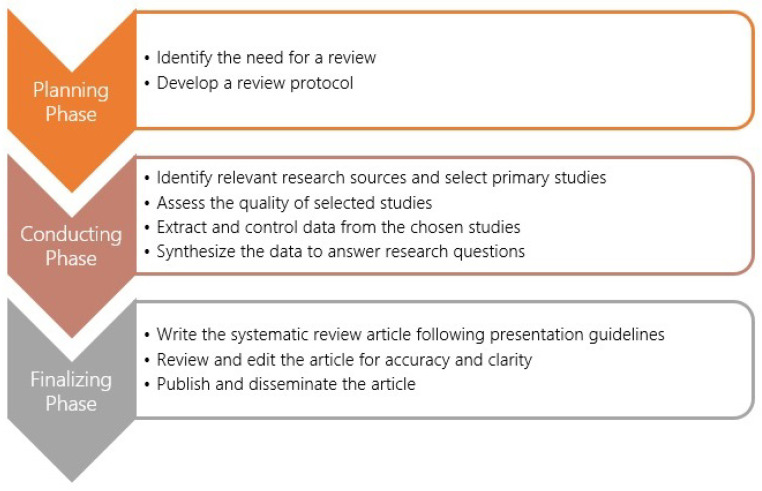
The overall systematic review process.

In conducting this systematic review, we followed the established methodology outlined by [19], with adaptations to suit the specific requirements of this study. The review process (see Figure 1) began with the planning phase, which involved identifying the need for a review and developing a review protocol. This was followed by the conducting phase, which entailed identifying relevant research, selecting primary studies, assessing study quality, extracting and controlling data, and synthesizing the data to answer the research questions. The final step of the process involved preparing the review report, which included writing the systematic review article in accordance with presentation guidelines; reviewing and editing the article; and, finally, publishing and disseminating the article.

**Figure 2:**
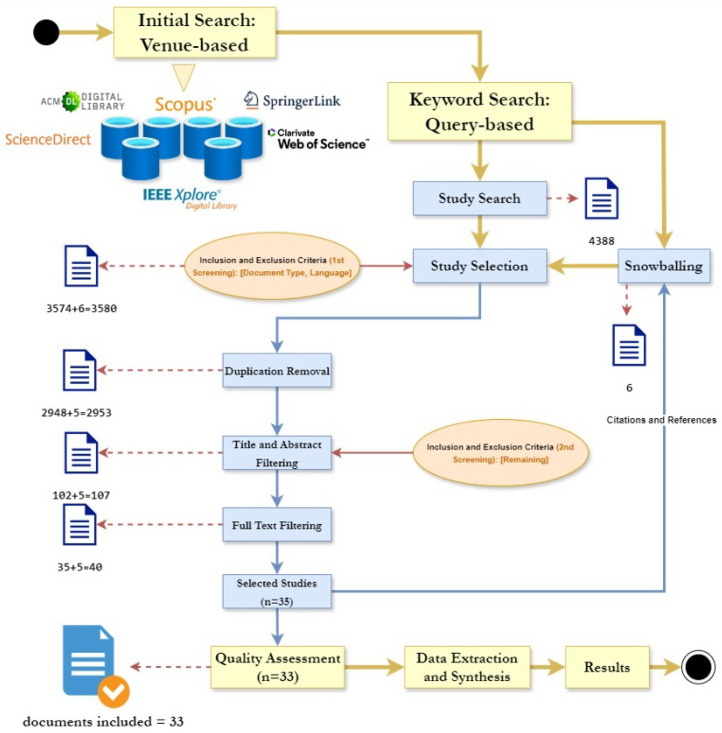
The proposed research framework for the conducting phases of the systematic review.

Figure 2 illustrates the methodology employed in the conducting phase of this research to ensure comprehensive coverage of relevant literature on the use of secure microservices for IoT.

#### 2.3.2. Time Frame and Keywords

The review process commenced on 1 April 2024, focusing on studies published between 2010 and 2024. In the Web of Science (WoS) database, keywords related to microservices, IoT, and security were used, including variations and synonyms to ensure comprehensive coverage. The search employed the “TS” field to encompass titles, abstracts, and keywords, and the “NEAR” operator refined results by ensuring key terms appeared close to each other. This approach yielded 136 results initially, narrowed to 78 with proximity searches, and further refined to 55 relevant studies. For other repositories, similar keywords and wildcard characters were used to capture a broad range of relevant literature. The asterisk (*), for instance, is used as a wildcard to expand searches by including different variations of a word’s root. For example, searching for “security” would return results including “secure”, “securing”, etc., providing a broader range of relevant articles. This strategy aimed to balance specificity and inclusivity, ensuring thorough coverage of the topic and minimizing irrelevant results.

#### 2.3.3. Search Results

As illustrated in Figure 2, the investigation commenced with a systematic identification of the repositories that would be subjected to comprehensive search and analysis. The selected repositories included esteemed scholarly platforms such as WoS, ACM Library, IEEE Xplorer, Scopus, SpringerLink, and ScienceDirect. Following this initial venue-based exploration, the focus shifted towards the identification of pertinent keywords, as elucidated in Table 1. Each keyword string was thoughtfully tailored to align with the unique indexing and retrieval mechanisms of the respective databases. Subsequently, concerted efforts yielded a substantial collection of 4388 publications encompassing the aforementioned databases, with SpringerLink emerging as the repository housing the highest number of retrieved papers.

The distribution of papers across all consulted databases is visually represented in Table 2, shedding light on the varying contribution of each repository to the research corpus.

#### 2.3.4. Studies Selection

The following inclusion and exclusion criteria were defined in order to collect primary studies and reports from the literature. The following are the inclusion criteria:

- Studies related to microservice-based IoT systems;
- Studies whose primary focus is security aspects of microservice-based IoT systems;
- Studies that provide solutions, methodologies, security mechanisms, or other procedures to handle microservice-based IoT systems;
- Studies written in English, French, or Arabic.

The following are the exclusion criteria:

- Short studies (less than 5 pages for academic literature);
- Secondary or tertiary studies (such as literature reviews, surveys, and others) *;
- Tutorial papers and editorials;
- Grey literature;
- Studies without full text available;
- Studies that do not link microservices and IoT;
- Not accessed electronically.

In a systematic review, the focus is on analyzing original research studies to provide the highest level of evidence. Secondary or tertiary studies, like literature reviews or surveys, are valuable on their own but can introduce bias or duplication of evidence. To ensure the review’s rigor and reliability, these studies are excluded, allowing the review to concentrate on primary studies and maintain a clear and objective analysis of the available evidence.

#### 2.3.5. Selection Process

In this study, we utilized Mendeley [20], a reference management software, to efficiently organize and manage the references from all the studies. Mendeley’s user-friendly interface and deduplication feature helped the authors identify and eliminate 626 duplicates from an initial 3574 papers, resulting in 2948 unique references. These were then imported into Covidence [21], an online systematic review platform, which facilitated the selection process through title and abstract screening, reducing the dataset to 102 papers. Covidence’s intuitive interface and collaborative features allowed multiple reviewers to assess and filter studies based on predetermined inclusion and exclusion criteria. Further examination of introductions, conclusions, and full texts narrowed down the dataset to 35 final candidates. Using forward and backward snowballing techniques, we identified six additional papers, with one duplicate, leading to a final set of 40 papers. This rigorous process ensured the accuracy and integrity of the reference list, making it suitable for subsequent quality assessments.

#### 2.3.6. Quality Assessment

The quality assessment is a crucial step in conducting a systematic review, as it ensures the reliability and validity of the included studies. To streamline and structure the assessment process, we have chosen to present the criteria as a series of questions in a weighted table format. Each question is carefully crafted to address specific methodological aspects of the studies, such as study design, data collection methods, and findings. By assigning weights to each question, we are able to prioritize the criteria based on their relative importance in contributing to the overall quality of the studies. The weights were determined on a numerical scale ranging from 1 to 3, with 1 denoting a moderate level of importance, 2 representing a high level of importance, and 3 signifying a significantly high level of importance. Commencing with a moderate level was a deliberate choice based on the rationale that each criterion selected for evaluation should possess a minimum moderate influence in order to justify its inclusion in the assessment process. This approach allows for a systematic and transparent evaluation of the included studies, facilitating the synthesis of evidence and enabling us to draw robust conclusions based on the most rigorous and reliable research available.

The questions included evaluating studies based on their alignment with research objectives, relevance to IoT microservice security, and methodological clarity. They assess the appropriateness of evaluation metrics, research design, and the effectiveness of proposed solutions for IoT security. Insights into security issues and vulnerabilities deepen understanding of the field while examining the practical applicability and feasibility of security mechanisms in real-world IoT contexts providing a comprehensive view of each study’s value. Additionally, citation count and publication venue offer insights into the studies’ academic recognition and impact. The cumulative score for each study is obtained through the summation of its measured values. The maximum achievable score is 28, while the average score across the included studies is 14. In order to ensure the inclusion of only high-quality studies, those with a quality score equal to or exceeding 14 are selected for incorporation into this study. See Table 3.

#### 2.3.7. Data Extraction Form

Data extraction was performed by identifying pertinent information (refer to Table 4) from the selected studies in response to the systematic study and its eight primary research questions. To streamline the synthesis process, we employed Covidence to create a data extraction template and store the discrete information.

## 3. Results and Analysis

### 3.1. Quality Assessment Results

The search was performed in April 2024, encompassing a time frame from 2010 to 2024; however, the earliest discovered publications originated from 2015 onwards, thereby forming the basis of the study’s scope from 2015 to April 2024. In order to evaluate the quality of each study, a set of predefined criteria was established, documented in Table 3. In instances of divergent assessments, collaborative meetings were conducted to reach a consensus on the final decision. Consequently, the resulting compilation of articles consists of 33 entries, labeled P1 (Paper1) to P33 (Paper33), as shown in Table 5.

Table 5 provides a comprehensive summary of the key studies selected for this systematic review. Each entry in the table includes the publication type, publisher/conference, year, and quality assessment score. By organizing the references in this manner, readers can easily see the diversity and relevance of the sources analyzed to draw conclusions about the potential of microservices in IoT security. Additionally, the quality assessment scores offer insight into the rigor of each study, helping readers understand the criteria used for their inclusion in this review.

### 3.2. Results Discussion

The selected studies that will be incorporated into this systematic review cover a wide range of topics within the field of MSA IoT solutions, including collaborative deep-learning microservices for backdoor defenses in Industrial IoT networks, frameworks for automation in context-aware IoT systems, and security schemes for failure detection in microservices IoT-edge networks, among others. The publications span various years and were published in diverse outlets such as journals and conferences by publishers like IEEE, ACM, Elsevier, and Springer. Each study is accompanied by relevant metadata, including publication year, citations, and a score indicating its perceived quality or significance. A quick glance at the landscape of MSA IoT solutions research reveals several notable observations. Firstly, a majority of the publications come from journals rather than conferences, indicating a preference for detailed and in-depth analysis within the academic community. This preference suggests a higher emphasis on thorough exploration and validation of concepts in MSA IoT solutions. Secondly, there is a clear diversification of technologies incorporated with MSA in IoT systems, ranging from deep learning and blockchain to edge computing and fog computing. This diversification underscores the multidisciplinary nature of MSA IoT research and highlights the need for integrated approaches to address complex challenges in IoT ecosystems. Additionally, only a limited number of studies exclusively target the raw security of IoT systems, with many focusing on broader aspects such as automation, interoperability, and performance analysis. This observation suggests potential opportunities for further exploration and innovation in enhancing the security aspects of MSA IoT solutions. Overall, this compilation of studies will serve as a valuable resource for this systematic review, providing insights into the evolving landscape of MSA IoT solutions and highlighting key advancements and trends in the field.

### 3.3. Number of Publications per Year (RQ1)

The number of publications per year is a crucial indicator of the research activity and interest in the domain of microservices in IoT. By analyzing the publication trends, we can gain insights into the growth and development of the field. Periods of increased research activity can highlight emerging topics and innovations, while a steady increase in publications may indicate a growing recognition of the importance of the field.

**Figure 3:**
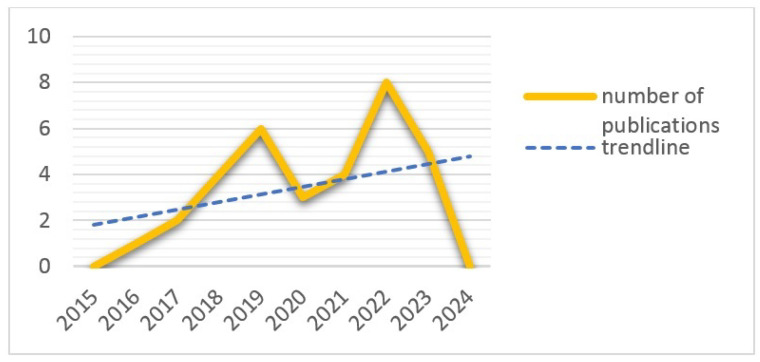
Number of publications per year.

**Figure 4:**
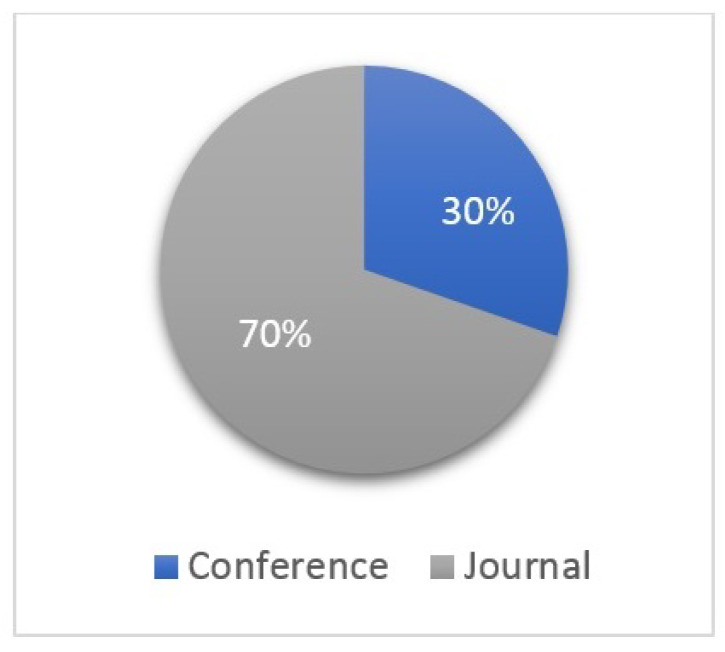
Publication venues.

Figure 3 illustrates the trend of publications in the field over the years, showcasing a consistent increase in interest and research activity in MSA IoT. The number of publications began to rise in 2016 and continued to grow steadily thereafter. Particularly noteworthy was the significant surge in publications in 2022, indicating heightened interest and research in the MSA IoT domain. However, the lack of publications in 2024 within the field of MSA IoT solutions, despite the previous upward trend, could be attributed to several factors. These may include the potential saturation of research topics, a shift in research focus towards other emerging areas, economic or institutional influences affecting funding and priorities, or methodological challenges impeding progress. Nonetheless, this temporary slowdown in research output does not necessarily imply a decline in the importance or relevance of MSA IoT solutions but rather reflects natural fluctuations in research activity. It is worth mentioning that most papers in the field are published in journals, as indicated in Figure 4.

The increasing number of publications overall suggests that MSA IoT remains an evolving and dynamic area of research, garnering growing attention from both academic and industry communities. Understanding these trends can help researchers and practitioners identify potential gaps and opportunities for further investigation.

### 3.4. Primary Publication Outlets (RQ2)

**Figure 5:**
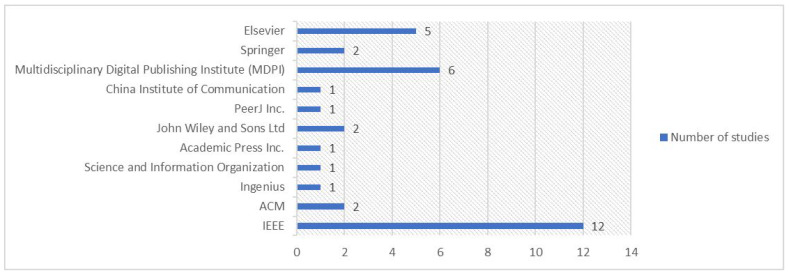
Primary publication outlets.

Classifying the styles and locations of publications on a specific topic can prove highly beneficial for researchers working on related subjects. The distribution of studies on microservice-based IoT systems across different publishing venues reveals that IEEE has the highest number of publications with 12 papers (see Figure 5). MDPI and Elsevier follow with six and five publications, respectively, and several other publishers have contributed one or two papers. This diverse representation indicates a growing interest and research focus on microservice-based IoT systems in various academic and industry circles.

### 3.5. Benefits of MSA in IoT (RQ3)

The assortment of benefits outlined below has been meticulously extracted from the comprehensive array of studies that were carefully selected and thoroughly reviewed during the course of this analysis. These selected studies encompass a diverse range of research efforts from various fields (see Table 6), including agriculture and the industrial IoT (IIoT), dedicated to investigating the advantages of adopting microservices architecture in IoT systems. Through this rigorous examination, we have identified and compiled these valuable insights, which shed light on the immense potential and promising outcomes that can be achieved by integrating microservices into the intricate landscape of IoT applications.

- **Flexibility and Scalability**: Microservices enable the ability to scale specific parts of the application as needed, making it possible to execute services on resource-constrained devices and efficiently manage resources.
- **Technology Heterogeneity**: Microservices allow different parts of the application to be implemented with diverse technologies, providing flexibility and adaptability in the IoT ecosystem.
- **Interoperability and Enhanced Performance:** Microservices facilitate seamless integration of diverse devices and services, fostering interoperability within IoT ecosystems. This architecture not only streamlines communication between components but also enhances performance by allowing independent scaling of services. As a result, microservices empower IoT applications to respond swiftly and effectively to user needs, ultimately improving system reliability and responsiveness.
- **Continuous and Easy Deployment**: MSA promotes continuous deployment practices, allowing for rapid updates, bug fixes, and feature enhancements, leading to more agile and responsive IoT applications.
- **Resource-Constrained Device Support**: MSA’s flexibility allows specific microservices to run on resource-limited IoT devices, enhancing the capability of IoT systems in various deployment scenarios.
- **Agile Development Process Owing to MSA’s Modular Design**: With microservices, IoT applications benefit from an agile and flexible development process, allowing rapid deployment and adaptation to changing requirements.
- **Resource Optimization and Management**: With MSA, resources can be allocated and managed more effectively, resulting in optimized performance and reduced wastage of computing resources.
- **Real-time Data Processing**: Automated microservices frameworks enable real-time processing of big data, improving fog resiliency and ensuring high-quality service delivery in IoT-Fog-Cloud ecosystems.
- **Fault Tolerance and Anomaly Detection**: In MSA, failures in one microservice do not affect the entire application, as each service operates independently, leading to improved fault isolation and system resilience.
- **Secure Authentication and Authorization**: Microservices architectures offer efficient authentication and authorization mechanisms, safeguarding IoT applications against unauthorized access and ensuring reliable user management.
- **Privacy and Data Protection**: Microservices architectures ensure secure communication channels, data encryption, and access control mechanisms, enhancing privacy and safeguarding against data breaches.
- **Enhanced Security**: Integrating other technologies like Blockchain technology into microservices architecture provides decentralized data management and robust security mechanisms, ensuring data integrity and access control in IoT applications.

Building upon the inherent benefits of microservices architecture in IoT, the presented Table 7 demonstrates how the adoption of MSA has become instrumental in accommodating a diverse array of security measures within the resource-constrained IoT ecosystem. Given the limitations of IoT devices in terms of computational power, memory, and energy resources, integrating robust security mechanisms presents a challenge. Nevertheless, the strategic implementation of MSA allows for the distribution of security functionalities across multiple microservices, effectively managing the resource load and optimizing overall system performance. Table 7 provides a concise and well-organized compilation of various security mechanisms, accompanied by pertinent examples extracted from primary studies.

While the table’s list is non-exhaustive, its value lies in the systematic grouping of security mechanisms alongside their respective papers, shedding light on any potential inclinations towards specific security measures favored by MSA IoT designers. Such insights enable researchers and practitioners to comprehend prevalent security practices in MSA-based IoT solutions and provide a basis for further exploration into emerging security mechanisms relevant to this context.

**Figure 6:**
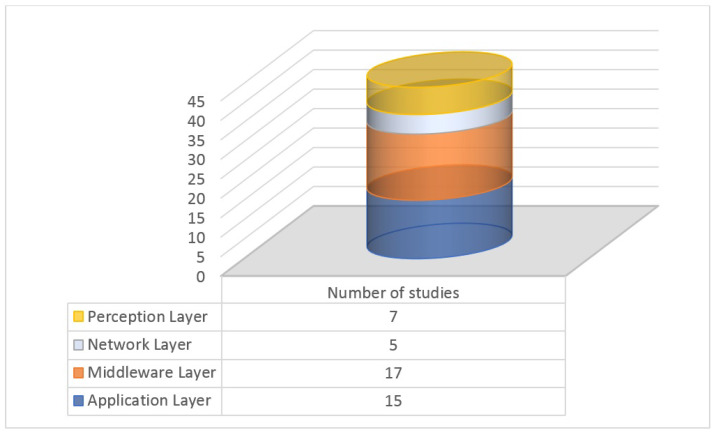
IoT architectural level of MSA security mechanisms applied in MSA IoT solutions.

The data presented in Figure 6 show the distribution of research studies concerning the applicability of security mechanisms across different layers of the IoT architecture. As per the figure, the middleware layer has the highest number of studies (17), indicating a significant focus on security mechanisms at this level. The application layer (15 studies) and the perception layer (7 studies) follow closely. The network layer has the fewest number of studies (5) in this context. It is important to note that the number of studies does not necessarily reflect the complexity or significance of security mechanisms within each layer. However, this information provides a valuable glimpse into the researchers’ interest in exploring and applying security solutions across various architectural IoT levels. Further analysis and research would be required to understand the specific trends and implications of these findings in the context of MSA IoT or other related fields.

**Figure 7:**
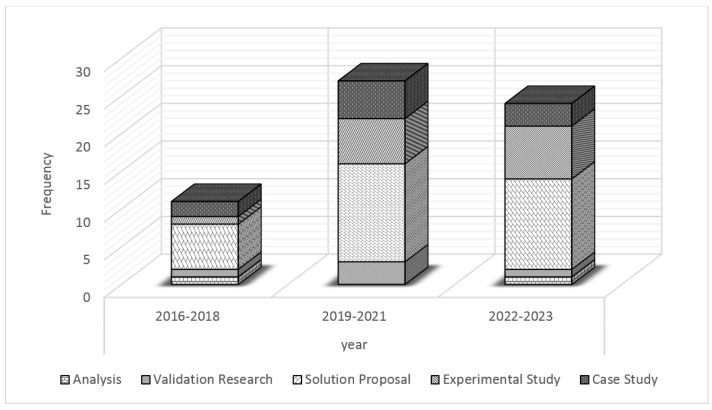
MSA IoT research activities over the years (2016–2023).

Another valuable insight that can be gleaned from the compilation of studies is a concise overview of research activities concerning IoT systems and microservices from 2016 to 2023 (see Figure 7). Notably, there is a consistent focus on solution proposals, with a steady increase observed over the years. The number of case studies fluctuates, indicating researchers’ interest in real-world implementations and performance assessments. Additionally, experimental studies show a clear upward trend, reflecting a growing inclination toward empirical evaluations of microservices in IoT systems. Overall, the table highlights the continuous efforts and rising interest in exploring the integration of microservices to enhance IoT applications.

Among the solution proposals, the prevalence of frameworks and algorithms solutions (P1, P2, P4, P9, and P21) indicates a strong focus on creating adaptable frameworks supported by innovative algorithms. Architecture (P3, P6, P11, P12, P17, P18, P23, P24, P29, P30, P32, and P33) also emerges as a prominent theme, showcasing the importance of designing robust and scalable systems. Some papers delve into the development of platforms and interfaces, providing a solid foundation for future implementations. Moreover, the inclusion of techniques (P16 and P27) and tools (P19) emphasizes the interdisciplinary nature of technology research. This diverse range of solutions demonstrates the dynamic and multifaceted nature of technological advancements, offering a glimpse into the variety of approaches employed by researchers to drive innovation and shape the future of technology.

Furthermore, an endeavor was undertaken to classify the IoT security needs addressed in the primary studies into potential security needs and standard security needs. Subsequently, each need was mapped to its corresponding paper. In literature, standard security needs encompass fundamental requirements for ensuring confidentiality, authentication, authorization, integrity, and attack defense. These security needs are widely recognized and implemented to provide a baseline level of protection for various systems and applications. On the other hand, potential security needs refer to advanced requirements that go beyond the basic security aspects. These needs focus on achieving scalability, efficient data management, fault tolerance, real-time data currency, and other advanced features that may be essential for specific applications or systems. By addressing both standard and potential security needs, organizations can create comprehensive security measures that cater to different levels of threats and vulnerabilities.

**Figure 8:**
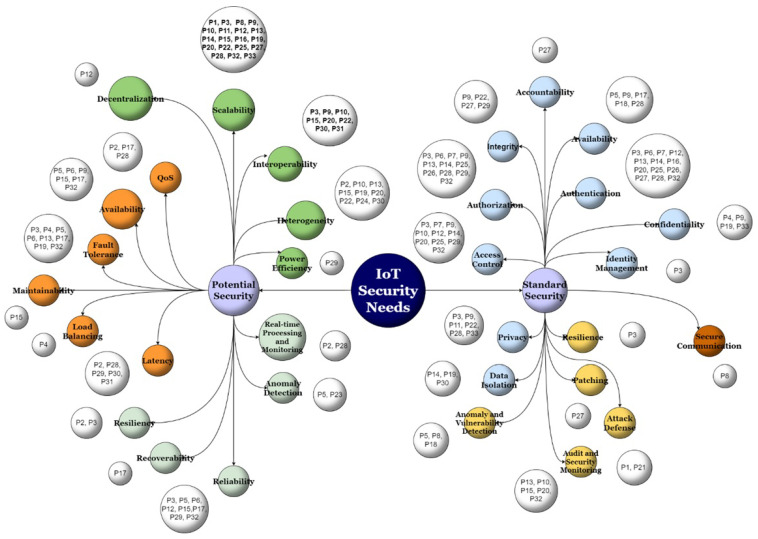
Mapping of IoT security needs with corresponding papers.

Figure 8 illustrates a preference for potential security needs, indicating that a considerable number of architecture designers prioritize leveraging this technology for performance enhancement rather than security requirements. Among the potential security needs discussed in the papers, scalability, interoperability, and heterogeneity emerged as dominant keywords, while authentication and authorization had the most significant share of standard security needs.

### 3.6. Key MSA IoT Challenges (RQ4)

While microservices offer an environment conducive to the implementation of security measures, they do not completely resolve IoT security challenges. Microservices enable better scalability and modular security implementation, but issues like secure communication between services and managing distributed components still persist. Other challenges include service discovery, data management, resource allocation, and deployment complexity. These obstacles must be addressed to ensure the effective implementation and operation of MSA in IoT environments. Complementary technologies can help bridge these gaps by providing tamper-proof mechanisms and enhanced data protection.

Table 8 provides a comprehensive discussion of the most significant challenges drawn from a selection of the studies.

While these studies highlight key obstacles, they do not provide explicit solutions. The proposed solutions in this section are self-suggested and derived from existing research and theoretical insights, to address the complexities discussed. These approaches offer practical considerations and strategies for overcoming the identified challenges.

### 3.7. Security Risks in MSA IoT (RQ5)

Extending the knowledge gained from exploring the challenges, the fifth research question examines the security risks associated with MSA in the IoT environment, specifically highlighting the potential threats and vulnerabilities arising from its adoption in IoT systems. Unauthorized access, data breaches, insecure communication channels, denial-of-service attacks, and compromised microservices are a few examples of these threats, discussed in Table 9.

To mitigate these security risks, it is important to implement secure coding practices, robust authentication and authorization mechanisms, encryption for data in transit and at rest, thorough input validation, container security measures, and proactive monitoring and logging to detect and respond to security incidents promptly. Regular security assessments and penetration testing can also help identify and address vulnerabilities in the microservice-based IoT system. While this systematic review focuses on the benefits of using MSA to enhance IoT security, it is also crucial to consider how to secure MSA-based IoT systems comprehensively. Some works, like [55], specifically address the strategies for securing these systems, emphasizing the need for a holistic approach to IoT security.

### 3.8. Performance Implications (RQ6)

When discussing the “implications” of something, we refer to the potential consequences or outcomes resulting from a specific action, decision, or situation. In the context of the performance implications of using microservices in IoT systems, it involves analyzing the effects on the system’s performance due to adopting a microservice architecture. Examining factors such as scalability, latency, network overhead, resource utilization, and complexity is pivotal to understanding their influence on the overall system performance. By considering these implications, informed decisions can be made, optimizations can be implemented, and potential trade-offs can be addressed to align the chosen architecture with the desired performance objectives and requirements.

The following aspects, derived from some of the studies encompassed in this research, merit consideration:

1. **Scalability**: Microservices offer the advantage of scalability, allowing individual components to scale independently based on demand. However, improper scaling strategies or excessive communication overhead between microservices can impact performance. Ensuring effective load balancing and efficient communication mechanisms is crucial to maintaining optimal performance.
2. **Latency**: Communication between microservices may introduce additional latency compared to monolithic architectures (P9 [30], P12 [33] and P28 [49]). Each request may need to traverse multiple services, potentially leading to increased network latency and response times. Proper design and optimization techniques, such as caching, asynchronous messaging, and event-driven architectures, can mitigate latency issues.
3. **Network Overhead**: Microservices communicate with each other over the network, leading to increased network traffic (P12 [33] and P28 [49]). This can cause network congestion and affect performance, particularly in resource-constrained IoT environments. Careful consideration of network protocols, data compression techniques, and efficient message-passing mechanisms can help manage network overhead.
4. **Service Discovery**: In a microservice architecture, services need to discover and communicate with each other dynamically. This introduces additional overhead for service discovery mechanisms, such as service registries or DNS resolution. The efficiency and performance of service discovery mechanisms can impact the overall system performance.
5. **Resource Utilization**: Microservices require additional resources, such as memory, CPU, and storage, compared to a monolithic architecture (P31 [52] and P28 [49]). The increased resource utilization can impact the performance of the underlying infrastructure, particularly in resource-constrained IoT devices. Proper resource management and optimization techniques are essential to ensure efficient resource utilization.
6. **Complexity**: Microservices introduce additional complexity in terms of managing and coordinating multiple services. The increased complexity can affect performance due to additional processing overhead and potential bottlenecks. Proper design and monitoring practices, along with effective management of dependencies, can help mitigate the impact of complexity on performance.

It is important to note that the performance implications of using microservices in IoT systems can vary based on the specific implementation, workload characteristics, and system requirements. Proper design, optimization, and performance testing are crucial to ensuring that the benefits of microservices outweigh any potential performance trade-offs.

### 3.9. Practical Aspects of Implementing Microservices in IoT Systems (RQ7)

After conducting an in-depth analysis of 31 studies pertaining to MSA IoT systems, a set of best practices has been extracted to provide valuable guidance for the effective implementation of MSA in IoT systems. These practices are intended to bolster security measures, enhance scalability, and optimize overall system performance.

1. Best Practice #1: System Decomposition and Security Considerations
  - *Description:* Breaking down large systems into smaller units of work (P25 [46]) and differentiating between various types of services (e.g., business services and infrastructure services).
  - *Implementation Guidance:*
    - Decomposing large, monolithic systems into individual microservices based on shared functionality, business logic, or domain boundaries, with Domain-Driven Design (DDD) helping to identify logical boundaries.
    - Using a zero-trust architecture by implementing strong authentication and authorization for each service and enforcing security policies at the service mesh level, ensuring secure communication.
    - Incorporating defense-in-depth by applying multiple layers of security, such as network- and service-level firewalls, and encrypting data both at rest and in transit.
2. Best Practice #2: Access Control and Identity Management
  - *Description:* Using identity providers to create and manage access tokens, enabling permission, profile, and credential management.
  - *Implementation Guidance:*
    - Granting limited access to external clients through firewalls and managing microservice access rights.
    - Delegating authentication (P24 [45]) to a single point and utilizing OAuth2 for lightweight identity infrastructure:
      (a) Delegated Authentication: OAuth2 allows microservices to offload authentication to a centralized identity provider. This reduces overhead by avoiding repetitive authentication processes in each microservice, which is especially beneficial in environments where resources are limited.
      (b) Token-Based Authorization: Once authenticated, the identity provider issues access tokens, which are short-lived and lightweight. These tokens are then used by the services to manage secure communication and resource access. Tokens ensure that services can authenticate and authorize requests without relying on resource-heavy identity checks, thus maintaining system efficiency.
      (c) For scalability, integrating rate limiting (restricting the number of requests a user or service can make in a time frame) and token expiration to prevent system overload, and centralizing access control through API gateways.
3. Best Practice #3: Scaling and Service Distribution
  - *Description:* Microservice architectures inherently allow horizontal scaling by replicating services to handle increased workloads.
  - *Implementation Guidance:*
    - Utilizing container orchestration platforms like Kubernetes or Docker Swarm for automated scaling, allowing services to adjust based on metrics such as CPU usage or memory consumption.
    - Applying horizontal pod autoscalers to dynamically manage scaling and distributing traffic evenly with load balancers across service instances.
    - Ensuring redundancy and fault tolerance by distributing services across nodes or clusters.
    - Implementing throttling (limiting the rate of request processing) and rate limiting at the API gateway level to prevent high traffic from overwhelming individual services.
4. Best Practice #4: Autonomous Development and Topology Mapping
  - *Description:*
    - Addressing new demands with individual microservices to maintain autonomous development.
    - Facilitating mapping service (P20 [41]) requests to microservice topologies to prevent rough dependencies and enhance scalability.
  - *Implementation Guidance:*
    - Developing, deploying, and scaling microservices independently through CI/CD pipelines, ensuring continuous integration and delivery.
    - Using service topology maps to visualize and organize relationships between services. Tools like service mesh can help map dependencies and communication paths, preventing bottlenecks and ensuring scalability.
5. Best Practice #5: Accountability and Certificate-based Security
  - *Description:* Establishing tracking measures for actions and adopting certificate-based solutions (P27 [48]) for IoT service authentication and integrity.
  - *Implementation Guidance:*
    - Implementing certificate-based authentication with mutual TLS (mTLS) for secure service communication, ensuring each service verifies the identity of others.
    - Managing digital certificates through Public Key Infrastructure (PKI), with certificate authorities automating certificate issuance and renewal.
    - Logging all service interactions and activities using auditing tools to track and trace responsibility across services.
6. Best Practice #6: Reusability, Safety, and Multi-microservice Requirements
  - *Description:* Reusing microservices across systems to improve efficiency and safety, especially in complex IoT environments.
  - *Implementation Guidance:*
    - Designing microservices with modularity to ensure reusability across various applications or domains, adhering to SOLID principles (e.g., the single responsibility principle), which promotes clean and maintainable code.
    - Avoiding dependencies between multiple microservices for single functionalities, which can lead to complex orchestration. Using event-driven architectures (e.g., Apache Kafka or RabbitMQ) to decouple services and enable asynchronous, safe communication.
7. Best Practice #7: Layer Segmentation and Security Scenarios
  - *Description:* Segmenting the IoT system into local (edge) and centralized (cloud) layers to improve security and performance.
  - *Implementation Guidance:*
    - Using edge computing for real-time data processing in local layers and offloading long-term storage and analytics to the cloud, ensuring performance and efficiency.
    - Implementing specific security measures at each layer, such as using edge computing gateways for localized processing and securing data transmission to the cloud with end-to-end encryption (e.g., TLS 1.3).
    - Applying role-based access control (RBAC) at each layer to ensure only authorized entities can access sensitive data.
8. Best Practice #8: Efficient Service Aggregation
  - *Description:* Using API gateways as single-entry points for aggregating microservices.
  - *Implementation Guidance:*
    - Deploying API gateways to handle incoming requests, routing them to appropriate microservices, and managing security features like rate limiting, caching, and request/response transformations.
    - For fine-grained requests (requests for specific, smaller pieces of data), using API gateways that support GraphQL or gRPC, which allow clients to specify the exact data they need. Coarse-grained requests (larger, bulkier requests) can be aggregated efficiently, as well, to reduce response times and improve system performance.

By categorizing the best practices, we can gain a better understanding of their common themes and applications, making it easier to implement and optimize microservices in IoT systems effectively.

### 3.10. Future Directions for Research (RQ8)

Through a comprehensive examination and synthesis of the selected studies, this investigation has yielded a myriad of compelling results and profound insights, which engender promising trajectories for future endeavors in the domain of microservices adoption within IoT systems.

#### 3.10.1. Limited Emphasis on Standard Security Measures

The study uncovered an interesting pattern in the literature on microservice IoT systems’ security. Most articles focused on potential security issues, such as scalability and interoperability, with approximately 13% of them exclusively addressing these concerns. These challenges relate to the ability of microservice architectures to handle increasing workloads, adapt to dynamic changes, and integrate diverse IoT devices and platforms seamlessly. Conversely, the review reveals a relative scarcity of attention granted to standard security issues, including pivotal facets such as attack defense and mitigation. It is imperative to recognize the vital role played by information security mechanisms in ensuring the legitimacy and identity of both IoT devices and microservices. While potential security issues undoubtedly hold significance, the limited exploration of standard security concerns exposes a noteworthy research gap. Addressing these concerns, including robust authentication protocols, effective attack mitigation techniques, and secure communication channels, stands as an indispensable requirement for achieving comprehensive security and resilience within microservice-based IoT systems. Consequently, further investigation and research within these realms will undeniably contribute to the development of more secure and trustworthy architectures for microservice IoT deployments.

Most of the research on microservice-based IoT systems focuses on scalability and interoperability, while standard security measures like attack defense, mitigation, and secure communication receive limited attention. Future work must not only address this gap but also develop practical approaches for implementing these measures in real-world IoT systems. For example, integrating robust authentication protocols and secure communication frameworks in resource-constrained IoT environments may encounter performance trade-offs. The implementation of lightweight encryption techniques or multi-factor authentication could enhance security without compromising efficiency. Success in this area could be measured through real-world trials, such as deploying secure microservice architectures in industries like healthcare or smart cities, where data privacy is paramount.

#### 3.10.2. Inadequate Attention to Microservice-Based Security in IoT: Dominance of Machine Learning Approaches

A significant portion of the initial search results focused on securing IoT using machine or deep learning techniques. Articles on this topic dominated the literature, indicating a prevailing trend in the research landscape. However, the research community has yet to fully leverage the potential of microservices in securing IoT systems. While machine learning approaches have garnered significant attention, there remains a gap in exploring how microservices can enhance security in the IoT context. By capitalizing on the modularity, scalability, and flexibility of microservices, researchers can develop innovative security solutions tailored specifically for microservice-based IoT architectures.

While machine learning approaches for IoT security dominate current research, there is untapped potential in leveraging microservices for security enhancements. Future studies should explore **specific** use cases where microservice-based solutions outperform or complement machine learning methods. For instance, modular security services such as real-time threat detection and access control mechanisms can be built using microservices, which can be deployed, updated, and scaled independently. Challenges may arise in balancing microservice granularity with the overhead introduced by deploying multiple services, especially in low-power IoT environments. Real-world case studies from industries like automotive or logistics could provide valuable insights into the effectiveness of these solutions.

#### 3.10.3. Insufficiency of Security Patterns for Microservice-Based IoT Systems

The lack of published security patterns tailored to microservices in the academic literature [11], particularly in the context of IoT environments, represents a significant research gap. While some efforts, such as P10, have incorporated security as part of a generalized framework, there is limited exploration of comprehensive security measures within microservice architectures. Notably, the work P16 stands out as a specific contribution addressing the security of microservice-based IoT systems. This finding underscores the opportunity to propose effective security solutions for authentication, access control, communication security, and attack surfaces within these architectures. By addressing these challenges, researchers can make valuable contributions toward the development of more robust and secure microservice-based IoT systems, ensuring data integrity, confidentiality, and resilience. It is evident that further research is needed to bridge this gap and advance the state of security in microservice-based IoT systems.

The scarcity of comprehensive security patterns in the literature indicates a gap that future research can address. These patterns should be tailored to handle the dynamic and distributed nature of microservice-based IoT systems. Implementing microservice-specific security protocols, such as service mesh architectures for secure communication, presents an opportunity to strengthen IoT deployments. However, challenges related to interoperability and scalability will need to be tackled. Researchers should test these patterns in practical deployments across different industries, evaluating their performance in diverse IoT environments such as smart manufacturing or autonomous vehicles, where real-time security is critical.

#### 3.10.4. Integrating Blockchain and AI/Machine Learning Solutions

**Figure 9:**
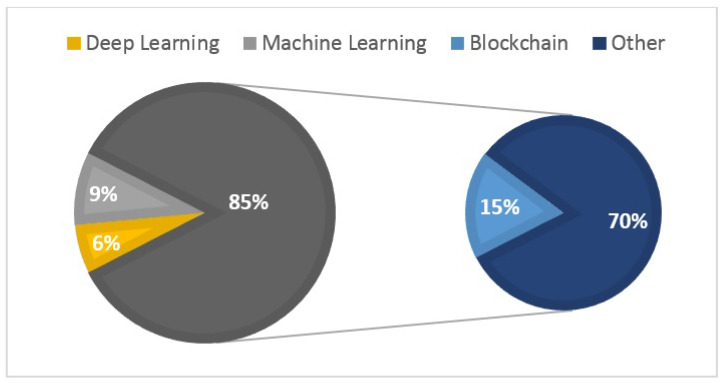
Distribution of technologies coupled with MSA.

Implementing blockchain integration is a promising future direction to enhance the security of microservice-based IoT applications. Leveraging blockchain features like tamper-proofing and robust encryption can improve trust and security. However, challenges such as scalability and energy efficiency need to be addressed. Blockchain as a Service (BaaS) offers a solution by providing a dynamic platform for IoT applications to utilize blockchain features seamlessly. Additionally, AI/ML-based solutions can enhance IoT security by identifying and predicting potential threats through decentralized learning systems. Several studies (P1, P2, P5, P8, P3, P9, P11, P12, and P13) included in this investigation have already examined the advantages of incorporating such technologies in conjunction with MSA for IoT systems, as depicted in Figure 9. Notably, 16% of the studies examined the combination of Blockchain technology with MSA, while Machine Learning was coupled in 10%, and Deep Learning in 3%. The rest of the studies (71%) focused on integrating MSA with various other technologies, encompassing domains like edge computing, big data, and specific industry-related technologies.

Blockchain integration offers a promising direction for improving the security and traceability of microservice-based IoT systems. However, challenges such as scalability and energy consumption must be addressed to make blockchain viable in practical IoT environments. Future research should investigate energy-efficient consensus mechanisms and scalable blockchain architectures. Additionally, Blockchain-as-a-Service (BaaS) could streamline blockchain integration for IoT applications. Field testing in sectors like supply chain management or smart energy grids would reveal the practical benefits and potential pitfalls of this integration. On the other hand, AI is increasingly being leveraged to enhance IoT security, with machine learning models employed to detect anomalies, predict cyber threats, and automate response mechanisms. The latest advancements include the use of federated learning, where AI models are trained across decentralized devices without sharing sensitive data, thus preserving privacy while improving security. Future research should explore how AI can be integrated with microservices to provide real-time, adaptive threat detection and response mechanisms, particularly in large-scale IoT deployments. Additionally, explainable AI (XAI) is emerging as a critical area, enabling more transparent and accountable decision-making processes in AI-driven security applications.

#### 3.10.5. Energy Efficiency and Sustainability

Given the critical concern of energy consumption in IoT systems [46], optimizing energy efficiency is paramount. Future research can delve into energy-aware microservice design, where microservices are optimized to minimize energy usage. Additionally, investigating energy-efficient communication protocols, power management strategies, and resource allocation algorithms for microservices in IoT will contribute to sustainable and energy-saving IoT deployments. Techniques like energy harvesting, dynamic power scaling, and energy-aware scheduling should be explored to achieve better energy utilization and reduce environmental impact.

Optimizing energy efficiency in microservice-based IoT systems is crucial for sustainability. Future work should focus on developing energy-aware microservice architectures and power management strategies. The use of dynamic power scaling and energy harvesting techniques can reduce energy consumption, but achieving these goals requires experimenting with prototypes in real IoT applications such as smart homes or agriculture. These real-world trials will help assess the feasibility and success of energy-efficient designs, especially in systems where battery life is critical.

#### 3.10.6. 5G/6G Technologies for Resource Allocation

The adoption of 5G and 6G technologies can significantly enhance resource allocation in microservice-based IoT systems. These advanced communication technologies offer high bandwidth, low latency, and support for massive device connectivity, enabling the efficient real-time processing and decision-making that is crucial for IoT applications [53]. By leveraging edge computing and network slicing, 5G and 6G can reduce latency, optimize resource utilization, and ensure the quality of service for various microservices. Research in this domain should focus on dynamic resource management, secure communication protocols, and scalable architectures, harnessing the capabilities of 5G/6G to create more efficient, reliable, and secure IoT systems.

The advent of 5G and 6G presents opportunities to enhance resource allocation in microservice-based IoT systems. By harnessing edge computing and network slicing, researchers can create architectures that optimize performance in real time. However, integrating these technologies with IoT microservices introduces challenges related to latency management and secure resource allocation. Practical case studies in areas like smart cities or connected vehicles will provide insights into how these technologies can be applied and what potential challenges may arise.

#### 3.10.7. Distributed Learning and Security-by-Design

Future research in the IoT domain can explore distributed learning-based and distributed data analytics-based approaches to improve resource utilization and system resilience. Extending intelligent functions through microservices, backed by trained prediction models, can revolutionize IoT applications for diverse industrial domains. A notable example of this is the work “iRECOVer: Patch your IoT on-the-fly”, P18 [39], a novel solution for next-generation IoT devices that follows the “Security-by-Design” principle, ensuring reliability and operational integrity throughout the IoT component lifecycle. Further investigation into microservices-specific security patterns is vital for robust security in IoT applications.

Distributed learning approaches can enhance the scalability and resilience of IoT systems. Future research should explore how microservice architectures can be designed with Security-by-Design principles, ensuring that security is embedded into each microservice from the outset. Implementing these designs in high-stakes environments like industrial control systems or healthcare could reveal the practical difficulties in maintaining security throughout the system’s lifecycle.

#### 3.10.8. Advancing Security Monitoring Techniques for Microservice-Based IoT Systems

There is a notable lack of specialized monitoring techniques for MSA-based IoT systems. Conventional solutions struggle to handle the complexity and dynamic nature of these architectures, leading to potential blind spots in security analysis. Developing dedicated monitoring tools is crucial for proactive threat detection and ensuring the security of MSA-based IoT deployments. This may involve creating tools and methodologies that efficiently analyze logs, network traffic, and system behavior within the context of MSA to identify potential security issues, anomalies, and malicious activities.

Specialized security monitoring techniques are needed to address the complexity of microservices in IoT systems. Developing dedicated monitoring tools that analyze system logs, network traffic, and behavioral patterns will be crucial for the early detection of anomalies. Implementing these tools in real-world systems like smart factories or IoT-enabled logistics chains would provide valuable data on their effectiveness and scalability. Potential challenges include managing data overload and ensuring that the tools can handle the distributed nature of microservices.

#### 3.10.9. Exploring Quantum Computing for IoT Security

While none of the studies selected in this review addressed quantum computing, this emerging field holds significant potential for enhancing the security of microservice-based IoT systems. Quantum computing presents a groundbreaking avenue for enhancing the security of IoT systems, particularly in the context of microservices. With its ability to solve complex problems exponentially faster than classical computing, quantum technologies could potentially revolutionize encryption mechanisms. The use of quantum key distribution (QKD) could strengthen communication channels between microservices by ensuring unbreakable encryption. However, the challenges of scalability, hardware availability, and the integration of quantum systems with current IoT and microservices infrastructure remain significant hurdles. Future research should focus on testing the feasibility of quantum-resistant cryptographic algorithms in microservice-based IoT environments, exploring their potential to protect against the looming threat of quantum-enabled cyberattacks.

### 3.11. Summary of Key Findings and Implications

The systematic review delves into the intersection of microservices and IoT systems, revealing several critical insights. We observed a significant increase in publications (RQ1) over the past decade, indicating growing research interest and investment in microservices for IoT systems. High-impact journals and leading conferences in computer science and engineering, are the primary publication outlets (RQ2), suggesting these as key sources for high-quality information. The study identified key benefits of microservices (RQ3), including improved scalability, flexibility, maintainability, and enhanced security through isolated services. These findings underscore the potential of microservices to significantly enhance IoT system performance and reliability. However, major challenges (RQ4) such as integration complexity, performance overhead, and the need for robust orchestration were also identified, with proposed solutions including advanced orchestration tools, lightweight containerization, and efficient service discovery mechanisms. Security risks (RQ5), including inter-service communication vulnerabilities and data breaches, were addressed with strategies like secure communication protocols and robust authentication mechanisms. Performance optimization remains crucial due to latency introduced by service interactions, with techniques such as efficient load balancing and caching being essential (RQ6). Best practices for implementation (RQ7), such as modular design, using lightweight containers, and adopting CI/CD pipelines, were highlighted to ensure successful and sustainable deployments. Future research directions (RQ8) include AI-driven service orchestration, edge computing integration, and advanced security frameworks, pointing towards opportunities for further innovation. Building upon the key findings of this paper, the following implications highlight the broader impact of the research:

- Theoretical Implications: the findings contribute to the growing body of knowledge on microservices and IoT, providing a detailed understanding of the benefits, challenges, and best practices.
- Practical Implications: practitioners can leverage these insights to improve the design, implementation, and management of IoT systems, leading to more robust and efficient solutions.
- Policy Implications: policymakers and industry leaders can use these findings to develop standards and guidelines that promote the secure and efficient adoption of microservices in IoT environments.

## 4. Microservice-Based IoT Systems Security Pattern Taxonomy

The fundamental objective of the following taxonomy is to contribute to the process of identifying, elaborating, and systematically classifying the microservice-based IoT systems security patterns presented in this revision. In this context, “security patterns” are reusable and proven solutions that address various security challenges and considerations within the context of IoT systems using microservices as the architectural approach.

This taxonomic classification was conceived in response to a notable deficiency observed within the academic literature. To the best of our knowledge, no existing studies have discussed Microservice-based IoT system security patterns. Although general security concerns and practices are addressed, a paucity of detailed, specialized, and comprehensive security patterns, tailored to the unique requirements of IoT systems employing microservices architecture, is evident. Recognizing this research gap, we initiated the development of a taxonomic classification of security patterns specifically designed for Microservice-based IoT systems, targeting the unique challenges of this architecture. These patterns are tailored to handle the decentralization of services with secure communication and data integrity, manage complex service interactions through robust API security, and support scalability with dynamic access control and policy enforcement. Additionally, they include fault-tolerant mechanisms to maintain security during failures, address microservice-specific threats with service-level encryption and logging, and manage identities and configurations securely.

**Figure 10:**
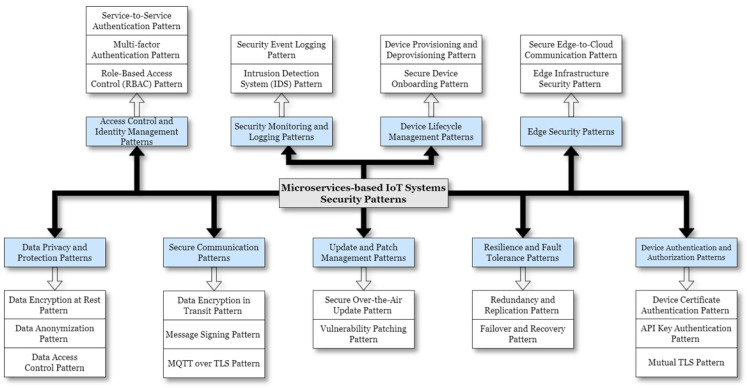
Taxonomic classification for MSA IoT security patterns.

While these patterns are specifically optimized for microservice-based IoT systems, we acknowledge that some of these solutions may also be applicable to other architectural contexts. Our focus remains on providing a structured framework for understanding, organizing, and implementing security measures in microservice-based IoT systems, but the insights and approaches outlined may offer valuable perspectives for other system architectures as well. See Figure 10.

Some common security patterns found in MSA-based IoT Systems’ Security Pattern Taxonomy may include the following:

- **Device Authentication and Authorization Patterns**: These patterns focus on authenticating and authorizing IoT devices before allowing them to interact with microservices. Techniques like device certificates, API keys, and mutual TLS can be included.
- **Secure Communication Patterns**: These patterns address securing communication between IoT devices and microservices to prevent data interception and tampering. Examples include encryption of data in transit, message signing, and secure protocols like MQTT over TLS.
- **Data Privacy and Protection Patterns**: These patterns involve safeguarding sensitive data collected and processed by IoT devices and microservices. Techniques like data encryption, data anonymization, and data access controls can be included.
- **Edge Security Patterns**: These patterns deal with securing edge devices and gateways in IoT systems, where data are processed and filtered before being sent to the cloud. This includes securing the edge infrastructure and ensuring secure communication between edge devices and microservices.
- **Access Control and Identity Management Patterns**: These patterns address managing user and service identities, access control policies, and authentication mechanisms in MSA-based IoT systems.
- **Resilience and Fault Tolerance Patterns**: These patterns focus on building resilient IoT systems that can withstand potential security incidents and recover from failures.
- **Security Monitoring and Logging Patterns**: These patterns involve implementing monitoring and logging mechanisms to detect and respond to security threats and incidents in MSA-based IoT systems.
- **Update and Patch Management Patterns**: These patterns deal with managing software updates and security patches for IoT devices and microservices to ensure they are protected against known vulnerabilities.
- **Device Life-cycle Management Patterns**: These patterns address the secure onboarding, provisioning, and decommissioning of IoT devices within the microservices architecture.

While the proposed security pattern classification system aims to provide a structured approach to securing microservices in IoT environments, further validation is needed to ensure its comprehensiveness and practical applicability. Future work could involve field testing the classification system in real-world IoT deployments, ideally in collaboration with industry partners. This would allow for an evaluation of its effectiveness in mitigating security risks, improving scalability, and enhancing overall system performance. Additionally, feedback loops may be established to gather insights from practitioners, developers, and system architects working with IoT microservices. Such feedback would focus on the practicality of the patterns, any gaps or challenges identified, and suggestions for improvement. Based on these data, iterative refinements to the classification system could include adjustments to the classification criteria, the introduction of new patterns, or the removal of redundant ones. Each iteration would be tested and validated to ensure that the system remains both accurate and user-friendly. Through continuous validation and refinement, the aim is to enhance the reliability and practical utility of the security pattern classification system, ensuring its effectiveness across a wide range of microservice-based IoT implementations.

This classification serves as a foundational resource, offering tried and tested solutions to common security challenges in MSA-based IoT systems. It encourages efficient and secure development practices while providing flexibility for customization to meet specific security requirements. As the landscape of IoT and microservices evolves, this taxonomy remains open to expansion, enabling continuous refinement and adaptation to emerging security demands.

## 5. Threats to Validity

Undertaking a systematic review necessitates a meticulous consideration of potential threats to the validity of research [56]. Guaranteeing the credibility, reliability, and generalizability of the findings is paramount to deriving meaningful insights for the academic community and practitioners.

### 5.1. Selection Bias

A key concern in the systematic review process is selection bias, which may inadvertently lead to the exclusion of relevant papers, compromising the completeness of the current representation. To mitigate this threat, a comprehensive literature search was strategically designed, following guidance from Kuhrmann et al. [57] and from Petticrew and Roberts [58]. Additionally, forward and backward snowballing techniques were utilized to identify additional relevant papers and avoid bias from search engines, ensuring a thorough exploration of the available research. Stringent inclusion and exclusion criteria were applied and collectively approved by the authors through in-depth brainstorming and voting sessions using Covidence [21], an advanced review management tool. This approach aimed to eliminate potential personal bias that could arise from the judgment and experience of the researchers involved. Only the articles approved by at least two of the authors were ultimately selected for this study.

### 5.2. Quality Assessment

With a commitment to robust evaluation, we developed a rigorous quality assessment framework inspired by the Critical Appraisal Tools of clinical research papers [59,60]. In designing the review process, we took the initiative to introduce the idea of adding weights to each question, reflecting our own ingenuity in a modest way. Careful consideration was given to each question’s impact on the review phase, with weights incorporated accordingly to ensure a nuanced and informed evaluation. To minimize bias, we leveraged Covidence, an advanced review management tool, facilitating collaborative filtering and space for open discussion on opinions and conflicts. All three authors actively participated in the assessment process, further enhancing the rigor of this evaluation.

### 5.3. Scope and Generalizability

The scope of this review may limit the generalizability of the findings to specific contexts or domains. To enhance the applicability of the conclusions, the research question and inclusion criteria were meticulously defined to ensure the review encompasses a diverse range of fields and security scenarios.

### 5.4. Reporting and Language Bias

Recognizing the significance of inclusivity, we actively sought studies published in various languages and regions. When necessary, translation assistance was sought, ensuring that language bias was minimized.

### 5.5. Time Frame Limitation

While we opted for a specific time frame (2010 to 2024) to focus on recent research, we acknowledge potential limitations in excluding earlier or more recent contributions. Despite this constraint, this work strove to capture the most relevant studies available.

### 5.6. Systematic Errors

Systematic errors during data extraction or synthesis could potentially impact the accuracy of the findings. To mitigate this risk, we have rigorously implemented a meticulous data extraction process, adhering to the guidelines set forth by Peterson et al. [61]. Furthermore, a peer review system involving all authors has been established, with the first author designing the data extraction form and facilitating thorough discussions with the second and third authors to ensure its accuracy and reliability.

## 6. Conclusions

In this comprehensive systematic review, we thoroughly investigated the security aspects of IoT systems through the lens of microservices. The analysis of selected papers published since 2016 revealed the prominent security needs addressed by MSA architectures in IoT solutions. Potential security concerns like scalability, interoperability, and heterogeneity garnered significant attention, while standard security requirements focused on authorization and authentication. We identified key challenges in contemporary MSA IoT research, including secure data distribution, integration of resource-constrained IoT devices in MSA, and service discovery and scheduling. Container and API vulnerabilities emerged as specific security risks due to the fundamental reliance on containerization and APIs in MSA. The study also highlighted a strong emphasis on securing the middleware and application layers, reflecting their critical roles in microservices’ interaction and data processing. This investigation further revealed a diverse range of industries utilizing MSA for IoT solutions, with verification and validation methods predominantly relying on performance analysis and case studies. However, we noted limited emphasis on standard security measures and insufficient attention given to microservice-based security in IoT. In response, we introduced a taxonomic classification for security patterns, providing a valuable guide for developers in their pursuit of secure MSA-based IoT system development.

Reflecting on the findings, MSA offers opportunities for scalability and flexibility in IoT system security. However, the lack of comprehensive exploration of standard security and potential vulnerabilities necessitates cautious deployment. Further investigations are encouraged to explore MSA’s utility in detecting and mitigating attacks, and more in-depth studies are warranted to address individual microservice and data exchange vulnerabilities, thereby fortifying the overall security posture of MSA-based IoT implementations. Moving forward, and building upon the insights gained from this systematic review, future research endeavors will be focused on investigating the seamless integration of Machine Learning techniques with microservices to bolster the security of IoT systems. Additionally, we intend to design and implement an Active Intrusion Detection System (IDS) dedicated to real-time monitoring and response to security threats within the microservices framework. Through these prospective research efforts, we aim to make substantial contributions towards the advancement of robust and secure IoT solutions within the context of microservices architecture.

## Figures and Tables

**Table 1:** Search keywords of the study.

Keywords String	Database	Queries
• Microservices, micro-services, MSA. • Internet of Things, IoT, smart devices. • Security, secure, intrusion, vulnerabilities.	WoS	TS = (“microservices” OR “micro-services” OR “MSA”) AND TS = (“Internet of Things” OR “IoT” OR “smart devices”) AND TS = (“security” OR “secure” OR “intrusion” OR “vulnerabilities”) AND PY = (2010–2024)Results: 136
		TS = ((“microservices” OR “micro-services” OR “MSA”) NEAR/100 (“Internet of Things” OR “IoT” OR “smart devices”) NEAR/100 (“security” OR “secure” OR “intrusion” OR “vulnerabilities”)) AND PY = (2010–2024)Results: 78
		TS = ((“microservices” OR “micro-services” OR “MSA”) NEAR/50 (“Internet of Things” OR “IoT” OR “smart devices”) NEAR/50 (“security” OR “secure” OR “intrusion” OR “vulnerabilities”)) AND PY = (2010–2024)Results: 55
	Other Repositories	(“microservices” OR “micro-services” OR “MSA”) AND (“Internet of Things” OR “IoT” OR “smart devices”) AND (“security*” OR “intrusion” OR “vulnerabilities*”)

**Table 2:** Consulted databases and results.

Repository	Search Results
WoS	55
Scopus	205
ScienceDirect	1273
SpringerLink	1786
ACM	722
IEEE	347
Total	4388

**Table 3:** Quality assessment criteria.

Quality Assessment Criteria	ID	Question	Weight	Score Legend
Research Question and Objectives	C1.1	Are the objectives of the study well -stated and aligned with its research question?	3	- 1 (yes) - 0 (no)
	C1.2	Is the research question clearly defined and relevant to IoT microservice security?	3	
Study Design, Methodology, and Evaluation	C2.1	Are the methods and techniques used to investigate IoT microservice security clearly described?	2	- 1 (Clear description) - 0.5 (Mostly clear description with minor gaps) -0 (Unclear description)
	C2.2	Are the evaluation metrics and performance measures appropriate for assessing the effectiveness and efficiency of the proposed solutions?	2	-1 (Appropriate) - 0.5 (Some appropriate metrics, but room for improvement) - 0 (Inappropriate/NA)
	C2.3	Does the study employ an appropriate research design for investigating IoT microservice security aspects, such as experimental studies or case studies?	3	- 1 (yes) - 0 (no)
Results and Findings	C3.1	Do the findings provide meaningful insights into the security issues, vulnerabilities, or threats in IoT microservice?	3	- 1 (Meaningful insights) - 0.5 (Findings provide meaningful insights, but some areas lack depth) - 0 (Lack of meaningful insights)
Analysis and Discussion	C4.1	Does the study clearly address any specific security issues in IoT microservice systems?	2	- 1 (yes) - 0 (no)
	C4.2	Are the limitations of the proposed approaches or studies discussed?	1	- 1 (Limitations discussed) - 0.5 (Some limitations discussed, but not comprehensively) - 0 (Limitations not discussed)
Practical Applicability	C5.1	Are the proposed security mechanisms or approaches feasible and applicable in real -world IoT deployments according to the authors?	3	- 1 (yes) - 0 (no)
	C5.2	Does the study provide practical recommendations or guidelines for securing IoT microservices?	2	- 1 (Detailed and validated solution) - 0.5 (Overview of solution or framework) - 0 (No clear solutions)
Citation and Quality of References	C6.1	Has the study been cited by other articles?	2	- 1 (yes) - 0 (no)
	C6.2	Has the study been published in ranked journals or conference proceedings?	2	- JRC for journals: - 1 (Q1 or Q2) - 0.5 (Q3 or Q4) - 0 (not ranked)

**Table 4:** Data extraction form.

ID	Data Field	Description	RQ
1	PID	Paper ID + Title + First author’s name.	
2	Year	Year of published paper.	
3	Type of publication	Conference, journal, workshop.	
4	Study field	Agriculture, smart city, computer science, other.	
5	Study design	Case study, experimental study, solution proposal, analysis, validation research, other.	
5	Security aspects	Security aspects addressed: potential security, standard security, both.	
6	Solution type	General measures, methodology, architecture, framework, algorithm, application, tool, other.	
7	Security mechanisms	Security mechanisms proposed or used. Whether the study is coupled with blockchain, machine learning, or other technologies.	
8	Applicability level	Architectural level where the security mechanisms are applied.	
9	Benefits of MSA in IoT	Benefits of adopting microservices in IoT, particularly in terms of security.	RQ3
10	Key MSA IoT challenges	Possible challenges could include issues related to performance, security, interoperability, complexity, or resource constraints.	RQ4
11	Security risks in MSA IoT	Potential security risks associated with using microservices in IoT systems.	RQ5
12	Performance implications	Performance implications of using microservices in IoT systems.	RQ6
13	Best practices	Practical aspects of implementing microservices in IoT systems.	RQ7
14	Future directions for research	Most promising future directions for research in the area of microservices adoption in IoT systems.	RQ8

**Table 5:** Selected studies.

PID	Reference	Type	Publisher/Conference	Year	Score
P1	[22]	Journal paper	Elsevier	2022	25
P2	[23]	Conference paper	IEEE/ACM	2019	14.5
P3	[24]	Conference paper	IEEE	2018	21
P4	[25]	Conference paper	Springer	2022	20
P5	[26]	Conference paper	IEEE	2018	26
P6	[27]	Conference paper	ACM	2019	16
P7	[28]	Conference paper	IEEE	2017	17
P8	[29]	Conference paper	IEEE	2018	26
P9	[30]	Journal paper	MDPI	2023	25
P10	[31]	Journal paper	China Institute of Communication	2017	18
P11	[32]	Journal paper	IEEE	2021	26
P12	[33]	Conference paper	IEEE	2019	19.5
P13	[34]	Journal paper	PeerJ Inc.	2022	22.5
P14	[35]	Journal paper	John Wiley and Sons Ltd	2021	14.5
P15	[36]	Journal paper	Elsevier	2022	22.5
P16	[37]	Journal paper	MDPI	2019	26
P17	[38]	Journal paper	Springer	2020	21.5
P18	[39]	Journal paper	Elsevier	2022	26
P19	[40]	Journal paper	MDPI	2020	24
P20	[41]	Journal paper	Elsevier	2023	17
P21	[42]	Journal paper	MDPI	2022	27
P22	[43]	Journal paper	Academic Press Inc.	2019	15.5
P23	[44]	Conference paper	IEEE	2016	19.5
P24	[45]	Journal paper	Ingenius	2021	21.5
P25	[46]	Journal paper	MDPI	2023	21.5
P26	[47]	Journal paper	IEEE	2020	21.5
P27	[48]	Conference paper	IEEE	2018	24
P28	[49]	Journal paper	IEEE	2022	21
P29	[50]	Journal paper	Science and Information Organization	2021	14
P30	[51]	Journal paper	John Wiley and Sons Ltd	2019	17.5
P31	[52]	Journal paper	IEEE	2022	15.5
P32	[53]	Journal paper	MDPI	2023	23.5
P33	[54]	Journal paper	Elsevier	2023	20.5

**Table 6:** Summary of research fields in MSA IoT studies.

Field	Number of Studies
Computing models (cloud, edge, fog)	9
IoT Security	7
Industrial Internet of Things (IIoT)	2
Smart IoT	5
Agriculture	3
Communication infrastructures	1
Healthcare	4
General/Agnostic	2

**Table 7:** Security mechanism used in MSA IoT solutions reported by the papers.

Security Mechanisms	Examples	PID
MSA coupled with Deep Learning and Machine Learning for Security	STRIP-based backdoor detection; CycleGAN-based trigger identification; Unlearning-based model mitigation.	P1
	The first microservice utilizes complex event processing for real-time data stream analysis; the second employs machine learning for proactive fault tolerance.	P5
	Anomaly Detection model; Data-Centric Discovery.	P8
MSA coupled with Blockchain for Security	Dynamic Service Replacement and Isolation Mechanism; Smart contracts.	P3
	Tamper-proof resistant scheme; Blockchain-based data verification; System schedulability.	P11
	Security Policy; Smart contracts; Decentralized Security Microservices.	P12
	Decentralized authentication; Role-Based access control (RBAC) model; Dynamic message transmission mechanism.	P13
Anomaly Detection and Monitoring	Fault-tolerant Microservices framework for real-time data stream analysis and proactive fault mitigation.	P5
	Automated anomalous behavior detection in surveillance videos.	P23
	Log monitoring	P32
Encryption techniques	Encryption–Decryption (RSA, DES, and AES) and dynamic service interaction to address privacy concerns.	P22
	Modified Fully Homomorphism Encryption.	P4
	Secure Boot and Flash Encryption; AES Key Management.	P28
	AWS Key Management Service (asymmetric encryption)	P32
	TLS Encryption.	P24
Containerization and technological independence	Docker Containerization; Lightweight container-based virtualization architecture for IoT service coordination.	P7, P9, P28, P30, P31
API Security	REST API Security; API Gateways; Protocol Conversion; Traffic Restriction.	P7, P8, P15, P25, P32
Edge Gateways	Security and isolated edge gateways; Edge Gateway Replication.	P26, P28
Authentication and Authorization	IAM; Single Sign-On and Federation; Access Control Policies; Mutual Authentication Credentials, OAuth2 access tokens.	P3, P10, P14, P15, P18, P24, P25, P26, P32
Certificates and Signatures	PKI certificates.	P24
	Certificate pinning; Chameleon signatures.	P16
	Security certificate.	P19
	X.509 version 3 certificates; Fully distributed certificate revocation; Short certificate lifetimes; and Automated certificate renewal.	P27
Patch Management	Firmware Updates; Patch Management.	P20
Load Balance	Load balancer for each microservice; Workload assignment strategy.	P4, P28

**Table 8:** Key challenges in microservices architecture for IoT.

MSA IoT Challenge	Brief	Proposed Solutions	PID
Secure data distribution	With the vast amount of data generated and exchanged within IoT systems, ensuring data privacy, confidentiality, and integrity becomes paramount. Microservice-based applications must verify the authenticity of each service involved in communication to prevent malicious misuse by hackers. Robust authorization and access control mechanisms are necessary to address security requirements in heterogeneous environments.	Implementing end-to-end encryption, using protocols such as TLS, ensures data confidentiality during transmission. Additionally, adopting robust access control mechanisms like OAuth2 and Zero Trust security models can enhance authentication and authorization processes. Identity management frameworks like OAuth2 can also be integrated to handle authorization securely and efficiently.	P20, P24, P32
Secure Integration of Resource-Constrained IoT Devices in MSA	The challenge lies in ensuring that the applications can run efficiently and effectively on resource-limited IoT devices while maintaining desired levels of performance, functionality, and security.	To address this, lightweight security protocols such as Datagram Transport Layer Security (DTLS) can be employed. Additionally, edge computing can be leveraged to offload resource-intensive tasks to nearby devices or servers, reducing the computational burden on resource-constrained devices. Microservices can be designed to operate in low-power modes, minimizing the energy consumption of IoT devices.	P8, P4
Discovery and scheduling of services	Microservice-based applications are composed of fine-grained, distributed, and independent entities. Ensuring smooth operations and effective utilization of resources requires dynamically identifying and allocating services based on demand, availability, and priority. This challenge involves managing the dynamic nature of Microservices Architecture in IoT settings, taking into account factors like limited computational resources, intermittent connectivity, and constrained devices.	Service orchestration tools, such as Kubernetes, can dynamically manage microservices, ensuring proper load balancing and resource allocation based on service demand. Service mesh architectures can also be used to enhance communication between microservices by automating discovery and providing traffic management.	P20, P16
Dynamic Configuration and Interface Flexibility	Adopting Microservices Architecture (MSA) for IoT solutions requires dynamically configuring and defining interfaces between modules. The heterogeneous nature of IoT ecosystems demands flexible module interactions, and using well-defined service interfaces is essential. However, current practices lack standardized methods for this purpose, necessitating innovative approaches to streamline interactions effectively.	Employing containerization technologies like Docker can help in standardizing microservice interfaces and promoting portability across different IoT environments. Additionally, adopting APIs and using open standards such as REST or gRPC for communication ensures flexibility and simplifies the process of integrating diverse modules in IoT ecosystems.	P18
Heterogeneity in Firmware Extraction for IoT Devices	IoT devices are highly diverse and heterogeneous, often featuring a wide range of architectures, protocols, and applications. Manually extracting firmware from such a large variety of available binary firmware becomes a labor-intensive and time-consuming task. This scalability issue can impede the efficient assessment of security vulnerabilities and updates for a vast number of IoT devices.	Automated tools for firmware extraction and analysis can speed up the process. Standardizing the firmware formats used by IoT devices can also reduce the complexity of this challenge. IoT-specific solutions like MUD (Manufacturer Usage Description) can be employed to standardize behavior expectations.	
Comprehensive Vulnerability Detection for Diverse IoT Deployments	In the MSA IoT ecosystem, detecting vulnerabilities comprehensively across the diverse range of deployed IoT devices poses a challenge. Many existing detection techniques focus on specific types of vulnerabilities, limiting their effectiveness in covering the full spectrum of potential threats in IoT systems with a blend of different protocols, architectures, and applications. Developing holistic and adaptable vulnerability detection methods becomes essential to ensure the security of diverse IoT deployments.	Comprehensive security platforms like Microsoft Azure Security Center or AWS IoT Device Defender provide real-time vulnerability assessments across a variety of IoT architectures. Machine learning models can also be trained to detect patterns of anomalous behavior, improving the detection of unknown vulnerabilities across diverse deployments.	
Achieving Seamless Integration in IoT with Microservices	The smart domain of IoT comprises various software and platforms collecting and processing data, requiring effective coordination and communication to fully leverage the benefits of Microservices Architecture.	Using middleware platforms or IoT gateways that act as intermediaries between different systems can simplify integration. Standards like MQTT (Message Queuing Telemetry Transport) can be utilized for efficient messaging between IoT devices and microservices, ensuring smooth data exchange.	P9
Management Complexity	The increased number of microservices can lead to management complexity, making it challenging to maintain a consistent and robust security posture across the entire system	Implementing service orchestration frameworks such as Kubernetes or using microservices management platforms can automate much of the complexity involved in managing large numbers of microservices. These tools also provide features for monitoring, scaling, and securing microservices, reducing the overall management burden.	Self-Suggested

**Table 9:** Security risks in microservices architecture for IoT.

Security Risks in MSA IoT	Brief	PID
Container Vulnerabilities	Microservices are often deployed within containers, such as Docker containers, but inadequate security measures or mismanaged configurations can lead to exploitable vulnerabilities. Outdated or unpatched container images may contain known vulnerabilities, posing risks to the microservices and enabling unauthorized access. Using Docker to orchestrate the cloud environment exposes various security risks, including internally deployed malicious applications, infected containers, and malevolent or semi-honest hosts. These risks emphasize the importance of implementing robust security measures to protect the integrity and safety of the containerized microservices in the cloud.	P21
Risk of Performance Bottleneck	As the number of microservices increases, the complexity of coordinating and orchestrating them grows. If not carefully designed and optimized, the communication and data exchange between microservices might become inefficient, resulting in increased latency and reduced responsiveness. Performance bottlenecks may occur due to issues like high network traffic, resource contention, or inefficient data transfers between microservices. Such bottlenecks can negatively impact the real-time processing capabilities of the IoT system and degrade its overall performance.	P12
Distributed Data Sharing/API Vulnerabilities	Each microservice typically has its own set of APIs that allow data exchange and communication with other services. If not appropriately secured, these APIs could become entry points for attackers to exploit and compromise the overall system’s security.Exposing APIs without proper authentication and access controls might enable unauthorized access to sensitive data or functionalities. Additionally, insufficient data validation and input sanitization in microservices’ APIs can lead to security vulnerabilities like injection attacks or data breaches. The distributed nature of microservices can make it challenging to maintain consistent and robust security measures across the entire system.	P12, P21
Distributed Denial of Service (DDoS) Attacks	Microservice architectures distribute functionality across multiple services, making them more susceptible to DDoS attacks. If a specific microservice is overwhelmed with malicious requests, it can impact the availability and performance of the entire IoT system, potentially causing service disruptions or outages	P7
Insecure Communication	Microservices typically communicate with each other over networks, making them susceptible to security vulnerabilities. If proper encryption and authentication measures are not implemented, sensitive data transmitted between microservices can be intercepted or tampered with, leading to data breaches or unauthorized access	Self-Suggested
Insider Threats	Microservices are typically developed and maintained by different teams or individuals. This distributed ownership introduces the risk of insider threats, where a malicious or compromised insider can exploit vulnerabilities or gain unauthorized access to sensitive data across multiple microservices.	
Software Vulnerabilities	Microservices may rely on third-party software libraries, and if these libraries have known vulnerabilities, they can be exploited by attackers to compromise the entire system.	

## Data Availability

The datasets can be found by the authors at request.
